# Usefulness of core needle biopsy for the diagnosis of thyroid Burkitt’s lymphoma: a case report and review of the literature

**DOI:** 10.1186/s12902-018-0312-9

**Published:** 2018-11-19

**Authors:** Stella Bernardi, Andrea Michelli, Deborah Bonazza, Veronica Calabrò, Fabrizio Zanconati, Gabriele Pozzato, Bruno Fabris

**Affiliations:** 1 Department of Medical Surgical and Health Sciences, Università degli Studi di Trieste, Cattinara Teaching Hospital, Strada di Fiume 447, 34149 Trieste, Italy; 2 Endocrinology Unit - Azienda Sanitaria Universitaria Integrata Trieste, Cattinara Teaching Hospital, Strada di Fiume 447, 34149 Trieste, Italy; 3 Pathology Unit - Azienda Sanitaria Universitaria Integrata Trieste, Cattinara Teaching Hospital, Strada di Fiume 447, 34149 Trieste, Italy; 4 Haematology Unit - Azienda Sanitaria Universitaria Integrata Trieste, Cattinara Teaching Hospital, Strada di Fiume 447, 34149 Trieste, Italy

**Keywords:** Thyroid, Burkitt’s lymphoma, Thyroid lymphomas, Core needle biopsy, Case report

## Abstract

**Background:**

Thyroid lymphomas are an exceptional finding in patients with thyroid nodules. Burkitt’s lymphoma is one of the rarest and most aggressive forms of thyroid lymphomas, and its prognosis depends on the earliness of medical treatment. Given the rarity of this disease, making a prompt diagnosis can be challenging. For instance, fine-needle aspiration (FNA) cytology, which is the first-line diagnostic test that is performed in patients with thyroid nodules, is often not diagnostic in cases of thyroid lymphomas, with subsequent delay of the start of therapy.

**Case presentation:**

Here we report the case of a 52-year-old woman presenting with a rapidly enlarging thyroid mass. Thyroid ultrasonography demonstrated a solid hypoechoic nodule. FNA cytology was only suggestive of a lymphoproliferative disorder and did not provide a definitive diagnosis. It is core needle biopsy (CNB) that helped us to overcome the limitations of routine FNA cytology, showing the presence of thyroid Burkitt’s lymphoma. Subsequent staging demonstrated bone marrow involvement. The early start of an intensive multi-agent chemotherapy resulted in complete disease remission. At 60 months after the diagnosis, the patient is alive and has not had any recurrence.

**Conclusions:**

Clinicians should be aware that thyroid Burkitt’s lymphoma is an aggressive disease that needs to be treated with multi-agent chemotherapy as soon as possible. To diagnose it promptly, they should consider to order/perform a CNB in any patient with a rapidly enlarging thyroid mass that is suspicious for lymphoma.

## Background

Thyroid nodules are an extremely common occurrence. It is estimated that up to 67% of the population has a thyroid nodule that could be detected by ultrasonography [1]. Thyroid cancer occurs in 7–15% of cases depending on risk factors such as age, sex, family history, and radiation exposure [2]. Thyroid lymphoma accounts for less than 5% of all thyroid cancers. Nevertheless, clinicians should know how to manage this extremely rare occurrence, as the prognosis of the most aggressive subtypes depends on the earliness of the diagnosis and the subsequent start of multiagent chemotherapy regimens.

Routine medical work-up of patients with thyroid nodules is based on the evidence that fine-needle aspiration (FNA), preferably performed under ultrasonographic guidance and with rapid on-site evaluation by a cytopathologist [3], is the most sensitive and cost-effective method to assess their nature and/or the need for surgery [2, 4–6]. By contrast, FNA has a low accuracy for the diagnosis of thyroid lymphomas [7, 8], often leading to diagnostic surgery. It has been argued that in cases suspicious of thyroid lymphomas, core needle biopsy (CNB) could help to reduce diagnostic surgery [7] and, most importantly, to obtain earlier the diagnosis necessary to start life-saving treatment with multiagent chemotherapy.

Here we report the case of a woman with Burkitt’s lymphoma of the thyroid gland, where CNB helped us to overcome the limitations of routine FNA cytology and to prescribe the right medical treatment. We also performed a review of the literature and a search in Pubmed of other clinical cases of adult patients affected by Burkitt’s lymphoma of the thyroid gland. For this purpose, we used the combined terms “Burkitt”, “thyroid”, and “case”, and we selected only English written articles [9–24], while we excluded a few reports in other languages, such as Spanish [25–28], French [29, 30], and Japanese [31, 32].

## Case presentation

**Fig. 1:**
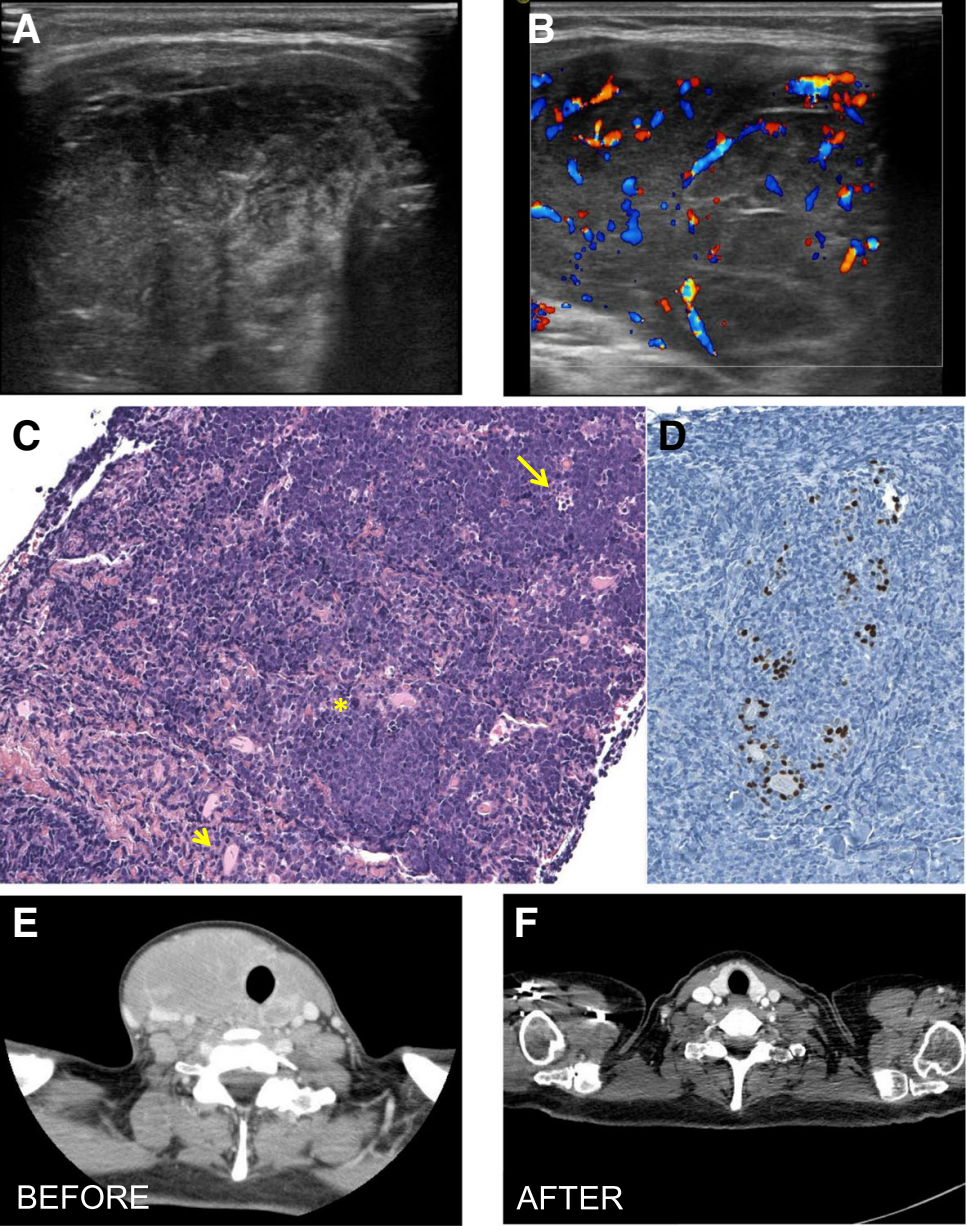
Endocrine imaging of a patient with Burkitt’s lymphoma. Thyroid US showed a solid hypoechoic mass (**a**) with significant internal vascularity (**b**) and no calcifications. Core needle biopsy showed homogeneous medium-sized B cells infiltrating the thyroid (**c**, arrowhead), mitotic figures (**c**, asterisk), and scattered macrophages ingesting apoptotic cells (**c,** arrow), giving a “starry sky” appearance. The extent of thyroid infiltration can be appreciated on (**d**) where follicular cells are only those positively stained for TTF1 (thyroid transcription factor 1). The CT scan performed before treatment showed a 44x43x87 mm thyroid nodule (**e**), which disappeared at the CT scan performed one year after treatment completion (**f**)

A 52-year-old woman presented to our Endocrinology Unit with a growing thyroid mass, which had enlarged so rapidly she had become unable to wear her motorcycle helmet in the weeks prior to her visit. She suffered from Hashimoto’s thyroiditis for which she was taking levothyroxine. There was no history of neck irradiation or family history of thyroid cancer. On examination, there was a large, firm thyroid nodule on the right side of the neck, without palpable cervical lymphadenopathy. TSH was 4.79 μU/mL with FT3 and FT4 within the reference range. Otherwise, there was only a mild thrombocytopenia. Thyroid ultrasonography showed a solid hypoechoic nodule in the right lobe of the gland, with significant internal vascularity and absence of calcifications (Figure 1). FNA cytology with rapid on-site evaluation of the material adequacy showed that there were only atypical lymphoid cells with no thyrocytes and the specimens were considered suggestive of a lymphoproliferative disorder but insufficient to make a diagnosis, such that a CNB was scheduled for the following day.

After checking the blood coagulation profile, the patient underwent a CNB, which allowed histological/morphological tissue analysis. This showed that normal thyrocytes were virtually all replaced by homogeneous medium-sized lymphocytes with scanty blue cytoplasm, round nuclei, coarse chromatin, and multiple small nucleoli. There were frequent mitotic figures and scattered macrophages ingesting apoptotic cells, giving to the tissue section the so-called ‘starry sky’ appearance (Fig. 1). Overall, these features were consistent with the presence of a thyroid Burkitt’s lymphoma, and further investigations were ordered to confirm the diagnosis and evaluate the disease extent. A CT of chest and abdomen showed the 44x43x87 mm thyroid nodule with left tracheal deviation (Figure 1) without other visible masses or lymph nodes. Bone marrow biopsy showed almost 100% lymphoid infiltration, consisting of a population of intermediate-sized blast-like cells, with prominent nucleoli, which were replacing all normal cells. These cells expressed CD10, CD20, and were negative for Bcl2, CD34, and TdT. Altogether these results led us to the final diagnosis of stage IV Burkitt’s lymphoma [33].

The patient was admitted to our hospital’s Haematology Unit and was successfully treated with 3 cycles of Hyper-CVAD chemotherapy (cyclophosphamide, vincristine, doxorubicin and dexamethasone) completed in five months. The thyroid mass disappeared (Fig. 1) and the platelets returned to baseline levels. At 60 months after diagnosis the patient is alive, and remains disease-free at regular follow-up.

## Discussion

Burkitt’s lymphoma is one of the rarest [34] and also most aggressive subtypes of thyroid lymphomas [11]. It is considered the fastest growing human tumor, with a cell doubling time of 24–48 h [33]. It arises from B cells, where a chromosomal translocation, more frequently t(8;14)(q24;q32) and less frequently either t(2;8)(p12;q24) or t(8;22)(q24;q11), leads to the deregulated expression of the oncogene C-Myc, which promotes cell cycle progression [35]. As a result, this lymphoma is characterized by the presence of monomorphic medium-sized B cells with a very high proliferation rate and increased apoptosis. To the best of our knowledge, 23 cases of thyroid Burkitt’s lymphoma have been described in the English medical literature [9–24] (Table 1). The majority of them (13 out of 23) were cases of Burkitt’s lymphoma with disseminated disease (stage III/IV). Among them, at least 5 patients (22%) died within the first 2 years of follow-up [11, 21, 22, 24] (Table 1). These were cases of age greater than 60 years, advanced disease, or disease onset complicated by cavernous sinus thrombosis (Table 1). Consistent with this, advanced age, poor performance status, advanced stage, and central nervous system or bone marrow involvement are considered the most relevant prognostic factors of a poor outcome in Burkitt’s lymphoma [35]. Therefore, starting chemotherapy as soon as possible is key for a complete response.

**Table 1:** Reported cases of thyroid Burkitt’s lymphomas

Authors (ref)	Age (y) Sex	Sites of involvement	Stage	Symptoms (S) Diagnosis (D)	Follow-up (mo)	Outcome
Thieblemont [9]	46 M	Thyroid, cervical and mediastinal nodes, bone marrow, stomach	IV	S: asymptomatic D: CNB	NA	NA
Iqbal [10]	6 M	Thyroid, right atrium, right ventricle, pericardium, abdominal masses, CNS	IV	S: thyroid enlargement, anorexia, weight loss, shortness of breath D: biopsy of suprarenal mass	NA	Alive, CR
Ruggiero [11]	40 F	Thyroid, other sites	IV	S: obstructive symptoms D: FNA + CNB	Died after 3 months	
Kalinyak [12]	53 M	Thyroid, bone marrow	IV	S: obstructive symptoms D: FNA + bone marrow	27	Alive, CR
Camera [13]	56 M	Thyroid, mediastinum, kidneys, right femur	IV	S: pathological fracture D: FNA + open surgery	NA	Reduction of all lesions
Kandil [14]	60 F	Thyroid and cervical nodes	I	S: obstructive symptoms D: FNA + incisional biopsy	NA	Succesfully treated after 1 cycle of CT
Yildiz [15]	31 M	Thyroid, cervical and jugulodigastric nodes	I	S: obstructive symptoms D: open surgery	6	Alive, CR
Bongiovanni [16]	72 F	Thyroid, cervical nodes, liver and skeletal lesions	IV	S: fever D: FNA + CNB	NA	NA
Mweempwa [17]	58 F	Thyroid	I	S: obstructive symptoms D: FNA + CNB	4	Alive, CR
Albert [18]	16 M	Thyroid	I	S: obstructive symptoms D: open surgery	NA	Alive, CR
Zhang [19]	8 M	Thyroid	I	S: obstructive symptoms D: open surgery	48	Alive, CR
Cooper [20]	14 M	Thyroid, lung, kidney and pancreas	IV	S: malaise, lethargy, weight loss D: FNA + OWB	36	Alive, CR
Alloui [21]	70 M	Thyroid	I	S: obstructive symptoms D: CNB	Patient died of septic shock after 17 days	
Quesada [22]	24 NA	Thyroid, cervical, aortcaval, preaortic, and paraortic nodes	III	S: obstructive symptoms 5/7 D: FNA 5/7 + either CNB 4/7, or open surgery 3/7	41	Alive, CR
	28 NA	Thyroid and cervical nodes	I		361	Alive, CR
	47 NA	Thyroid, cervical nodes, CNS	IV		25	Alive, CR
	45 NA	Thyroid	I		12	Alive, PD
	41 NA	Thyroid, cervical, pretracheal and retrocrural nodes, mediastinum, bone marrow	IV		113	Alive, CR
	49 NA	Thyroid, cervical and iliac nodes	III		Died after 12 months	
	19 NA	Thyroid, cervical, jugulodigastric nodes, lumbar vertebrae	IV		Dieta after 23 months	
Akshintala [23]	21 F	Thyroid and cervical nodes	I	S: obstructive symptoms D: CNB + incisional biopsy	NA	Alive, CR
Moghaddasi [24]	47 F	Thyroid and cervical nodes	I	S: diplopia D: incisional biopsy	Died after 30 days	
Claudi [45]	56 F	Thyroid, liver	IV	S: obstructive symptoms D: open surgery	NA	NA

CNB is for core needle biopsy; CNS is for central nervous system; CR is for complete remission; FNA is for fine needle aspiration; NA is for not applicable; PD is for persistent disease; OWB is for open wedge biospy

Unfortunately, given the rarity of this disease, making a prompt diagnosis can be challenging. The first aspect that should raise the suspicion of a thyroid lymphoma should be the presence of a rapidly growing goiter or nodule. It is estimated that 70% of patients with aggressive thyroid lymphomas complain of a rapidly expanding cervical mass that causes obstructive symptoms, such as dyspnea and dysphagia [8, 36]. In line with this figure, these symptoms were reported by 65% (15 out of 23) of patients with thyroid Burkitt’s lymphoma (Table 1). However, these symptoms are not specific and they might also be due to other conditions, such as anaplastic carcinoma or Riedel’s thyroiditis. Moreover, sometimes, the thyroid mass due to a lymphoma can be an incidental occurrence in patients with fever, malaise, weight loss, or hypothyroidism due to Hashimoto’s thyroiditis, as reported by [9, 16, 20]. Otherwise, there have been also a few reports of exceptional presentations such as a pathological fracture due to a secondary lytic lesion [13], and the onset of diplopia and headache due to a bilateral cavernous sinus thrombosis [24].

According to current guidelines [2, 6], ultrasonography is the first exam that should be performed in patients with a goiter or a thyroid nodule, and it should be generally followed by FNA cytology, whenever a solid thyroid nodule greater than 1-2 cm is detected. However, in case of a thyroid lymphoma, these procedures are often nondiagnostic. For instance, the ultrasound features of thyroid lymphomas, which include very low echogenicity, enhanced posterior echoes, increased vascularity, and lack of internal calcifications, are all aspecific [36]. In addition, as shown by the rapid on-site evaluation of our specimens, FNA cytology is often suggestive but insufficient to make a diagnosis of thyroid lymphoma. Apart from not providing adequate material, other pitfalls of FNA include the cytological similarities with thyroiditis and the high rate at which both pathologies occur simultaneously in the same gland, as 60–90% of lymphomas arise on a background of thyroiditis [36]. For these reasons, it has been argued that patients with suspected thyroid lymphomas require CNB or excision for diagnosis [8].

Tissue biopsies can provide the material necessary to assess tissue morphology and to perform a panel of immunostains, which should be the first aspects to evaluate when a Burkitt’s lymphoma is suspected [37]. In particular, typical morphological features of this lymphoma include the presence of homogeneous medium-sized lymphocytes with round nuclei, coarse chromatin and multiple small nucleoli, surrounded by a scanty blue cytoplasm with frequent small vacuoles and indistinct edges [38]. Another typical feature is the “starry sky” pattern [16], which is due to the presence of macrophages containing apoptotic tumor cells on a background of proliferating B cells. Then, to reach a final diagnosis of Burkitt’s lymphoma, immunohistochemical stainings should provide evidence that lymphomatous cells express CD19, CD20, CD10, and CD79a and no CD3, CD5, Bcl2, and TdT [37, 38]. Additional diagnostic criteria for Burkitt’s lymphoma include Ki67 positivity/proliferation index > 90%, light chain restriction, nuclear c-myc positivity at immunocytochemistry, and t(8;14)(q24;q32) by fluorescence in situ hybridization [38], as reported by [9, 11, 16, 17, 21–23] (Table 1).

Core needle biopsy (CNB) is an exam that is not routinely performed in the work-up of patients with thyroid nodules. This is ascribed to higher cost, technical requirements, and concerns about potential complications as compared to FNA [39]. In addition, also CNB can fail to diagnose follicular carcinomas [40], whose presence is diagnosed based on vascular invasion and/or capsular breakthrough [39]. Consistent with these issues, the AACE/ACE/AME guidelines suggest considering the use of CNB only in solid nodules with persistently inadequate cytology [6], and the ATA guidelines do not even recommend its use [2]. Nevertheless, our case reminds that CNB should not be dismissed as it can become extremely useful in cases of thyroid lymphomas, where it allows to obtain a specimen that is adequate for histological/morphological tissue analysis, as well as for other key diagnostic tests. This is supported also by our literature review showing that CNB (but no FNA) was able to provide the final diagnosis without additional exams [9, 11, 16, 17, 21–23] (Table 1). In particular, in the 23 reports of Burkitt’s lymphoma, FNA cytology was performed in 12 patients (52%) and was able to provide the diagnosis without core needle or open surgical biopsy only in one case [12] (Table 1). Overall, in the reported cases of thyroid Burkitt’s lymphoma, diagnosis was provided by core needle biospy (43%; 10 cases out of 23), open surgery (35%; 8 cases out of 23), incisional/open wedge biopsy (17%; 4 cases out of 23), rarely by FNA (1 case out of 23) [12] or biopsy of other sites of involvement, such as a renal mass (1 cases out of 23) [10] (Table 1).

In line with the concept that CNB should be the advised modality for thyroid lymphoma diagnosis, Sharma and colleagues have recently shown that CNB diagnostic sensitivity for detecting thyroid lymphomas is 93% [41]. This is in line with the results of Ha and colleagues, who found that CNB sensitivity for thyroid lymphoma was 94.7% with a positive predictive value of 100%, such that CNB was able to significantly reduce the rate of diagnostic surgery from 37.9 to 5.3%, as compared to FNA [7]. Interestingly, in a work comparing CNB to open surgical biopsy in patients with lymphoadenopathies, CNB turned out to have greater sensitivity for detecting malignancy, and it was also faster, cheaper and safer than the conventional surgical approach [42].

Having said that, current treatment of Burkitt’s lymphoma in adults is based on the delivery of short-duration, dose-intensive, multi-agent chemotherapy with minimization of treatment delays, and maintenance of serum drug concentrations over at least 48 to 72 h [35]. Some protocols, like the French LMB, the German BFM, and the CODOX-M/IVAC [43], have been adapted from pediatric regimens. Others, such the Hyper-CVAD regimen [44], which is the one we used, have been evaluated primarily in adults, but incorporate the principles found to be effective in pediatric populations [35]. Overall, with these regimens, 65 to 100% of patients achieve a complete response and 47 to 86% of patients maintain these remissions at least 1 year after treatment completion [35].

## Conclusions

This case describes a patient with thyroid Burkitt’s lymphoma, which is a rare and highly aggressive thyroid malignancy that requires a prompt diagnosis in order to start as soon as possible life-saving multi-agent chemotherapy. In particular, our case highlights the usefulness of CNB for the diagnosis of thyroid lymphomas and reminds clinicians to order/perform it in any patient with a rapidly enlarging thyroid mass that is suspicious for lymphoma.

## Abbreviations

AACE/ACE/AME: American association of clinical endocrinologists/Association of clinical endocrinologists/Associazione medici endocrinologi
ATA: American thyroid association
BFM: Berlin-Frankfurt-Münster
CNB: core needle biopsy
CODOX-M/IVAC: cyclophosphamide, vincristine, doxorubicin, high-dose methotrexate/ifosfamide, etoposide, and high-dose cytarabine
CVAD: cyclophosphamide, vincristine, doxorubicin, and dexamethasone
FNA: fine needle biopsy
FT3: free triiodothyronine
FT4: free thyroxine
LMB: lymphoma malignancy B
TSH: thyroid stimulating hormone
